# Thermopower, figure of merit and Fermi integrals

**DOI:** 10.1038/s41598-021-03760-4

**Published:** 2021-12-21

**Authors:** Patrice Limelette

**Affiliations:** GREMAN, UMR 7347 CNRS-INSA-Université de Tours, Parc de Grandmont, 37200 Tours, France

**Keywords:** Electronic properties and materials, Thermoelectric devices and materials

## Abstract

The thermoelectric efficiency accounting for the conversion of thermal energy into electricity is usually given by the figure of merit which involves three transport coefficients, with the thermopower, the electrical and the thermal conductivities. These coefficients can be defined at a semi-classical level as a function of Fermi integrals which only allow analytical approximations in either highly degenerate or strongly non-degenerate regimes. Otherwise, the intermediate regime which is of interest in order to describe high thermoelectric performance requires numerical calculations. It is shown that these Fermi integrals can actually be calculated and that the transport coefficients can be reformulated accordingly. This allows for a new definition of the figure of merit which covers all the regimes of interest without numerical calculations. This formulation of the Fermi integrals also provides a good starting point in order to perform a power expansion leading to a new approximation relevant for the intermediate regime. It turns out that the transport coefficients can then be expanded by revealing their high temperatures asymptotic behaviors. These results shed new light on the thermoelectric properties of the materials and point out that the analysis of their high temperatures behaviors allow to characterize experimentally the energy dependence in the transport integrals.

## Introduction

By allowing for the reversible conversion of heat waste into electricity, the thermoelectric effects has attracted a renewed interest in the past decades likely due to the context of the environmental concern and the growing number of complex materials^1,2^. This has motivated materials science in order to find out new compounds fulfilling the specific thermoelectric requirements^3,4^ as well as condensed matter physics which aims to connect the thermoelectric efficiency to materials microscopic characteristics through the transport coefficients^5^.

As early raised by Chasmar and Stratton^6^, the transport coefficients which characterize the thermoelectric efficiency can be written as a function of Fermi integrals in the frame of the semi-classical treatment of the Boltzmann equation within the relaxation time approximation^7^. This description can be extended by including different scattering mechanisms^8–12^, bipolar effects^9^, multiple bands and valley degeneracy^13–17^. If one considers for instance that the relaxation time, the velocity and the density of states vary as a function of the energy according power laws^18^ with the exponents $\theta$, $\nu$ and $\gamma$ respectively, then the electrical conductivity $\sigma$, the thermopower $\alpha$ and the Lorentz number $L=\kappa _e / \sigma T$, with the electronic thermal conductivity $\kappa _e$, can be defined as it follows^6,8–10^.

1 $$\begin{aligned} \sigma= &\; {} \sigma _{ E_0 } s F_{ s-1 }( \tilde{\mu } ) \end{aligned}$$

2 $$\begin{aligned} \alpha= &\; {} \frac{k_B}{q} \left( \frac{(s+1) F_s(\tilde{\mu }) }{ s F_{s-1}(\tilde{\mu }) } -\tilde{\mu } \right) \end{aligned}$$

3 $$\begin{aligned} L= &\; {} \left( \frac{k_B}{q} \right) ^2 \left[ \frac{s F_{ s-1 }(\tilde{\mu }) (s+2) F_{ s+1 }(\tilde{\mu }) - (s+1)^2 F_{ s }^2(\tilde{\mu })}{s^2 F_{ s-1 }^2(\tilde{\mu })} \right] \end{aligned}$$

with $s=(\theta + \nu + \gamma )$ and the Fermi integral $F_s( \tilde{\mu } ) = \int _0^{\infty } ~\frac{ x^s }{ e^{x-\tilde{\mu }} +1 } dx$ which is a function of the reduced chemical potential $\tilde{\mu } = \mu /k_B T$^18^. As previously emphasized, the parameter s accounts for the energy dependence in the transport integrals by assuming that the relaxation time, the velocity and the density of states vary as power law with the aforementioned exponents as defined below.

$$\begin{aligned} \tau _E = \tau _0 ( E / E_{\tau })^{ \theta } \quad v_{x,E}^2 = v_0^2 ( E / E_{v})^{ \nu } \qquad g_E = g_0 ( E / E_{g})^{ \gamma } \end{aligned}$$

Here, the characteristic energies $E_{\tau }$, $E_v$ and $E_g$, and the constants $\tau _0$, $v_0$ and $g_0$ have been introduced in order to focus on energy dependence. This formalism is general in the sense it describes both metallic and insulating regimes, and appears especially relevant as long as the density of states is a monotonic function of the energy^19^.

According to these definitions two kinds of approximation can be performed depending on the degree of degeneracy which lead to analytical expressions. In the strongly non-degenerate regime as in insulators characterized by a gap, the Fermi integrals can be simplified such as $F_s( \tilde{\mu }<<-1 ) \propto e^{ \tilde{\mu } }$. At the opposite in the highly degenerate regime, a Sommerfeld expansion allows to write $F_s( \tilde{\mu }>> 1 ) \approx \frac{\tilde{\mu }^{s+1}}{s+1} + s\frac{\pi ^2}{6}\tilde{\mu }^{s-1}$ as usual in metals. As highlighted by Fistul^10^, the most difficult case to approximate is the intermediate regime where the degeneracy is moderate with typically $| \tilde{\mu }|<< 1$ and which requires so far the use of tables or numerical calculation^20^. Thereafter, it is shown that the calculation of the Fermi integrals allows for an analytical redefinition of the transport coefficients which cover all the regimes of interest including the intermediate one. It is also demonstrated that the calculated form of the Fermi integrals provides a good starting point in order to perform a power expansion leading to a new approximation specifically relevant for the intermediate regime which connects the highly degenerate to the strongly non-degenerate ones. This new expansion is especially useful in order to characterize the asymptotic behavior of the transport coefficients reached in particular at high temperatures.

## Results

Below the Fermi integrals are first calculated by recognizing a special function and then the transport coefficients in Eqs. (1), (2) and (3) are redefined accordingly. These new definitions allow to reformulate the figure of merit *ZT* which accounts for the thermoelectric efficiency in the frame of the material quality factor formalism including a finite lattice thermal conductivity.

### Calculation of the Fermi integrals

In order to calculate the Fermi integrals $F_s( \tilde{\mu } )$, a high temperatures expansion of the Fermi factor can be first performed in power of $e^{-(x-\tilde{\mu })}$ as it follows:

$$\begin{aligned} (e^{x-\tilde{\mu }} +1)^{-1}= &\; {} e^{-(x-\tilde{\mu })} (1+ e^{-(x-\tilde{\mu })})^{-1} = e^{-(x-\tilde{\mu })} \sum _{k=0}^\infty (-1)^k e^{-k(x-\tilde{\mu })} = \sum _{k=1}^\infty (-1)^{k-1} e^{-k(x-\tilde{\mu })} \end{aligned}$$

Then, the Fermi integrals can be calculated quite straightforwardly by using this expansion.

4 $$\begin{aligned} F_s( \tilde{\mu } )= &\; {} \int _0^{\infty } ~\frac{ x^s }{ e^{x-\tilde{\mu }} +1 } dx =\int _0^{\infty } ~x^s \sum _{k=1}^\infty (-1)^{k-1} e^{-k(x-\tilde{\mu })} dx = \sum _{k=1}^\infty (-1)^{k-1} e^{k \tilde{\mu } } \int _0^{\infty } ~x^s e^{-k x} dx \nonumber \\= &\; {} \sum _{k=1}^\infty \frac{(-1)^{k-1}}{k^{s+1}} e^{k \tilde{\mu } } \int _0^{\infty } ~x^s e^{- x} dx = \Gamma (s+1) \sum _{k=1}^\infty \frac{(-1)^{k-1}}{k^{s+1}} e^{k \tilde{\mu } } = - \Gamma (s+1) Li_{s+1}(-e^{\tilde{\mu }}) \end{aligned}$$

The last line above relates the Fermi integrals to the special function $Li_{s}(x)=\sum _{k=1}^\infty \frac{x^{k}}{k^s}$ known as the Polylogarithm and the well-known Gamma function $\Gamma (s)$. In particular, one recovers easily the expansion of the Fermi integral $F_0( \tilde{\mu } )$ which can be anyway integrated directly by identifying that $\sum _{k=1}^\infty \frac{(-1)^{k-1}}{k} e^{k \tilde{\mu } }= ln \left( 1+ e^{ \tilde{\mu } } \right) = - Li_1 (-e^{\tilde{\mu }}) = F_0( \tilde{\mu } )$. It may be emphasized that the relation between the Fermi integrals and the Polylogarithm can also be demonstrated by using the recurrence formula between the Fermi integrals without using the Fermi factor expansion as shown in the Supplementary information.

### Definitions of the transport coefficients

The electrical conductivity $\sigma$, the thermopower $\alpha$ and the Lorentz number $L= \kappa _e/\sigma T$, with the electronic thermal conductivity $\kappa _e$, are usually defined in terms of Fermi integrals according Eqs. (1), (2) and (3). Now, they can also be related to Polylogarithm functions as introduced in Eq. (4), with the new notation $Li_{s}(-e^{\tilde{\mu }})=Li_{s}$.

5 $$\begin{aligned} \sigma= &\; {} - \sigma _{ E_0 } \Gamma (s+1) Li_{s} \end{aligned}$$

6 $$\begin{aligned} \alpha= &\; {} \frac{k_B}{q} \left( (s+1) \frac{ Li_{s+1} }{ Li_{s} } -\tilde{\mu } \right) \end{aligned}$$

7 $$\begin{aligned} L= &\; {} (s+1) \left( \frac{k_B}{q} \right) ^2 \left[ (s+2) \frac{ Li_{s+2}}{Li_{s}} - (s+1) \frac{Li_{s+1}^2}{Li_{s}^2} \right] \end{aligned}$$

These relations allow then easy calculations of the transport coefficients from the non-degenerate regime to the degenerate one. They can also be a good starting point in order to characterize the transport properties in more complex systems such as multiple bands materials for instance without implying sophisticated numerical computation. In addition, they are especially useful in order to perform approximations depending on the chemical potential. For instance in the strongly non-degenerate regime with $\tilde{\mu }<<-1$, the first term of the Polylogarithm expansion ($k=1$) is the leading one, $Li_{s+1}(-e^{\tilde{\mu }}) \approx -e^{ \tilde{\mu } }$, and one recovers the usual approximation for the Fermi integral $F_s( \tilde{\mu }<<-1 ) \approx \Gamma (s+1) e^{ \tilde{\mu } }$. Therefore, it follows that the approximate forms of the transport coefficients in the strongly non-degenerate regime are easily deduced below.

8 $$\begin{aligned} \sigma \approx \sigma _{ E_0 } \Gamma (s+1) e^{ \tilde{\mu } } \qquad \alpha \approx \frac{k_B}{q} \left( (s+1) -\tilde{\mu } \right) \qquad L \approx (s+1) \left( \frac{k_B}{q} \right) ^2 \end{aligned}$$

Beyond this approximation, the general relations 5, 6 and 7 are also interesting in order to reformulate the dimensionless figure of merit *ZT* characterizing the thermoelectric efficiency and which combines the thermopower, the electrical conductivity and the thermal conductivity. If the electronic part of the thermal conductivity is only considered, then $ZT= \alpha ^2/L$ and Eqs. (6) and (7) allow to define a purely electronic figure of merit. Nevertheless, in real materials the lattice thermal conductivity $\kappa _l$ needs to be taken into account and the figure of merit can be written as a function of the so-called dimensionless material quality factor $B= (k_B/q)^2 \sigma _{E_0} T/ \kappa _l$ originally introduced by Chasmar and Stratton^6^ and redefined more recently^21^.

9 $$\begin{aligned} ZT = \frac{\alpha ^2}{L + \frac{(k_B/q)^2}{B s F_{s-1}(\tilde{\mu })}} = \frac{\left[ 1- \tilde{\mu } \frac{Li_s}{(s+1) Li_{s+1}} \right] ^2}{\left[ \left( \frac{s+2}{s+1} \right) \frac{Li_s Li_{s+2}}{ Li_{s+1}^2} - \frac{Li_s}{ (s+1) B \Gamma _{s+2} Li_{s+1}^2} -1 \right] } \end{aligned}$$

**Figure 1:**
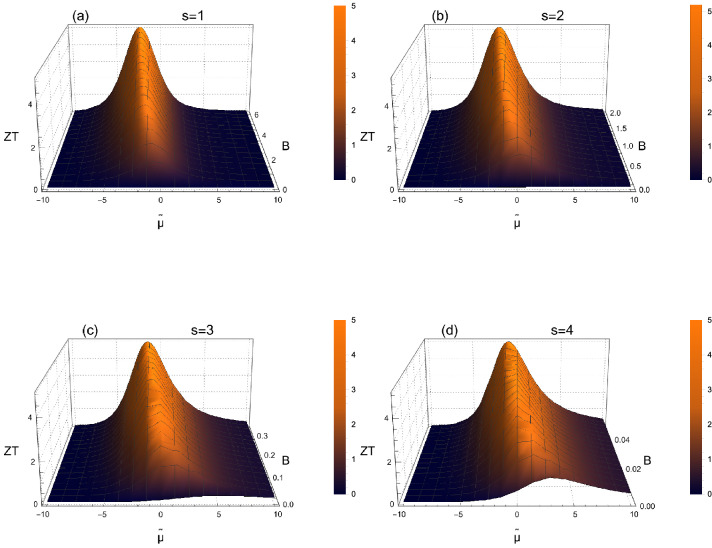
Figure of merit according Eq. (9) as a function of the reduced chemical potential $\mu /k_B T$ with $\hbox {s}=1$ (**a**), 2 (**b**), 3 (**c**) and 4 (**d**) for various material quality factors. The maximum of ZT shifts toward higher $\mu /k_B T$ and the range of high ZT-values increases if s is higher due the required lower B. The various exponents s from 1 up to 3 correspond to distinct scattering mechanisms while $\hbox {s}=4$ characterizes the expected behavior of Dirac-like quasiparticles scattered by unscreened ionized impurities in 3D.

The figure of merit defined in Eq. (9) allows then to plot straightforwardly its variations as a function of both the reduced chemical potential and the material quality factor as reported in Fig. 1 for selected exponents s.

For free electrons with a quadratic energy dispersion ($\nu = 1$) in three dimensions ($\gamma = 1/2$), it follows that $s = 3/2 + \theta$. If they are scattered by longitudinal acoustic phonons above the Debye temperature or by screened ionized impurities, then $\theta = -1/2$ and $s = 1$^10^. If they undergo polar scattering from optical phonons, then $\theta = 1/2$ and $s = 2$^21^, and if they are scattered by unscreened ionized impurities, then $\theta = 3/2$ and $s = 3$^18,22^. In addition, it has been shown that, for Dirac quasiparticles ($\nu = 0$) in three dimensions ($\gamma = 2$), the relaxation time due to scattering by unscreened ionized impurities varies quadratically as a function of energy, namely, $\theta = 2$ and then $s = 4$^18^. Actually, the latter exponent has been determined experimentally by several groups in a wide variety of conducting polymers^23–26^ which have been identified as promising thermoelectric materials^27–30^ due in particular to their low thermal conductivity^28,30,31^.

Therefore, the general formulations of the transport coefficients according to Eqs. (5), (6) and (7) allow us to investigate for different charge carriers, densities of states, and scattering mechanisms, the thermoelectric efficiency as a function of s varying from 1 up to 4 as reported in Fig. 1. As previously suggested, the exponent s ranging from 1 up to 3 corresponds to a single parabolic band dispersion ($E \propto k^2$) for different scattering mechanisms while the exponent $\hbox {s}=4$ is related to a single linear band dispersion ($E \propto k$). Whereas the chemical potential dependence of ZT is mainly characterized by a peak located around $\tilde{\mu } \approx 0$ in an intermediate regime between the non-degenerate and the degenerate ones, some specificities appear depending on the exponent s. In particular, the ZT-maximum is deeper located in the non-degenerate regime for low exponents s such as $\hbox {s}=1$, in contrast to $\hbox {s}=4$ for which the maximum shifts toward higher values of chemical potential including the slightly degenerate regime.

Additionally, the width of the ZT-peak significantly increases for larger s-values implying then that a better thermoelectric efficiency can be found in materials with quasiparticles characterized by strong energy dependence in the transport integrals, namely with higher exponent s. These variations also illustrate that lower material quality factors are required in order reach high ZT values for large exponents s suggesting that a larger lattice thermal conductivity may be sufficient in this case.

## Discussion

In order to compare the figure of merit with experimental results, it is necessary to introduce within this formalism the charge carriers density and to discuss the influence of the effective mass. Thereafter, the transport coefficients are more specifically considered in the intermediate regime and the related approximation is developed from the expansion of the Fermi integrals in power of the reduced chemical potential.

### Charge carriers density and effective mass

Whereas Eq. (9) allows to plot in Fig. 1 the figure of merit for distinct exponents s as a function of the reduced chemical potential, the latter is generally not measured experimentally and ZT is more usually determined as a function of the charge carriers density. Therefore, it is useful to relate the reduced chemical potential to the charge carriers density according Eq. (10) with the Fermi factor $f_E$ and the density of states such as $g_E=g_0 (E/E_g)^{\gamma }$, with a characteristic energy $E_g$ and the constant $g_0$.

10 $$\begin{aligned} n = \int _0^\infty g_E f_E dE = g_0 \frac{(k_B T)^{\gamma +1}}{E_g^{\gamma }} F_{\gamma }(\tilde{\mu }) = \frac{N_c(T, \gamma )}{\Gamma (\gamma +1)} F_{\gamma }(\tilde{\mu }) = - N_c(T, \gamma ) Li_{\gamma +1} (-e^{\tilde{\mu }}) \end{aligned}$$

**Figure 2:**
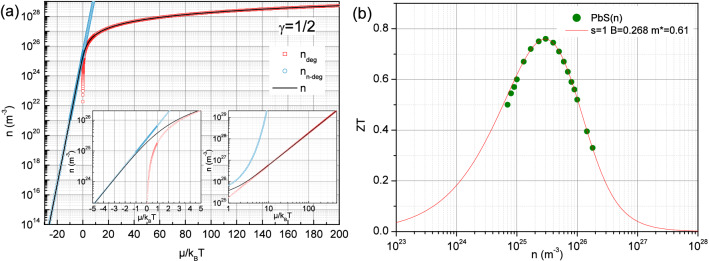
Charge carriers density according Eq. (10) with $\gamma =1/2$ as a function of the reduced chemical potential $\mu /k_B T$ at 300 K (**a**). The left inset displays the exponential behavior such as $n=N_c(T, \gamma )e^{\tilde{\mu }}$ in the non-degenerate regime and the right inset shows the power law behavior reached in the strongly degenerate regime as $n=\frac{1}{3 \pi ^2} \left( \frac{2 m \mu }{\hslash ^2}\right) ^{3/2}$. Comparison between the figure of merit determined experimentally in the n-type PbS compound at $\hbox {T}=800$ K with the one calculated from Eqs. (9) and (10) with $\hbox {s}=1$ and $\gamma =1/2$ (**b**).

**Figure 3:**
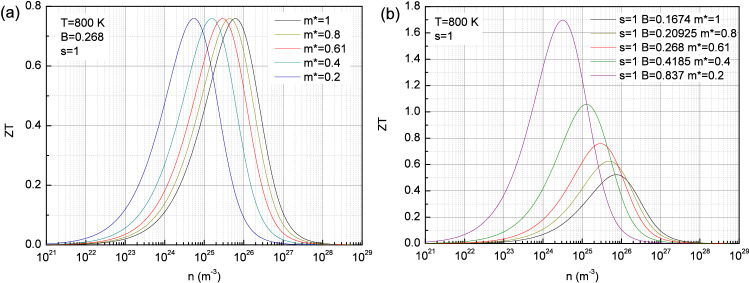
Variation of the figure of merit as a function of the charge carriers density at 800 K for selected low band effective mass with constant $\hbox {B}=0.268$ (**a**) and a material quality factor dependent on the effective mass as $B \propto 1/m^*$ (**b**). Here an isotropic system has been assumed with $m^*_b = m_I = m^*$.

Here, the temperature dependent constant $N_c(T, \gamma )$ has been introduced in order to recover the well-known exponential relation in the strongly non-degenerate regime such as $n=N_c(T, \gamma )e^{\tilde{\mu }}$^32^. The latter coefficient can be written as usual in the case of a free electrons density of states such as $N_c(T, \gamma =1/2) = \frac{1}{4} \left( \frac{2 m k_B T}{\pi \hslash ^2} \right) ^{3/2}$ whereas it acquires a different expression if one considers 3D Dirac-like quasiparticles with a parabolic density of states^18^ leading to $N_c(T, \gamma =2) = \frac{2}{\pi ^2} \left( \frac{k_B T}{ \hslash v_F} \right) ^{3}$, the latter being relevant in the case of the exponent $\hbox {s}=4$. By using Eq. (10) with $\gamma =1/2$, the charge carriers density can be plotted in Fig. 2(a) as a function of the reduced chemical potential at 300 K. In particular, one recovers here the two limiting behaviors with $n=N_c(T, \gamma )e^{\tilde{\mu }}$ in the non-degenerate regime and the power law followed in the strongly degenerate regime as $n=\frac{1}{3 \pi ^2} \left( \frac{2 m \mu }{\hslash ^2}\right) ^{3/2}$. Thus, Eqs. (9) and (10) allow to compare the expected variations of the figure of merit as a function now of the charge carriers density with the one measured experimentally in the n-type lead chalcogenide PbS at 800 K^21^ in Fig. 2(b). Whereas it seems that most of the other lead chalcogenides requires to take into account multiple bands effects^14^, the variations of the figure of merit of this compound appear well reproduced by simply adjusting the material quality factor and the effective mass. As discussed thereafter and displayed in Fig. 3(a), the latter constrained actually the charge carriers density corresponding to the maximum of the figure of merit and it is found to be 0.61 in Fig. 2(b) whereas it is known that the effective mass in PbS is 0.23 at 800 K^21^. Nevertheless, it is the overall density of states effective mass $m^*_d$ which determines the carriers concentration and can be related to a single band effective mass $m^*_b$ such as $m^*_d = N_v^{2/3} m^*_b$, with the valley degeneracy $N_v$^9^. In lead chalcogenides^21^, the valley degeneracy for the conduction band is $N_v = 4$ which leads to a single band effective mass $m^*_b \approx 0.24$ according the results in Fig. 2(b) in good agreement with the expected one. It is also worthy to mention that whereas the influence from polar scattering can be found at 300 K when the carriers density is lower than $10^{25}\,\hbox {m}^{-3}$, it decreases at higher temperatures such as 800 K compared with the acoustic phonon scattering which is even more predominant within this regime^21^.

In the other n-type lead chalcogenide PbSe for instance, the maximum of the figure of merit reaches nearly 1.13 at 800 K for a charge carriers density of the order of 1.9 $10^{25}\,\hbox {m}^{-3}$^21^, namely a higher value than in PbS in Fig. 2(b) for a lower charge carriers density. While this higher figure of merit appears consistent with the higher material quality factor $\hbox {B}=0.47$, the lower charge carriers density suggests at a qualitative level a lower density of states effective mass such as $m^*_d \approx 0.48$ by using both Eqs. (9) and (10). If one considers the same conduction bands valley degeneracy as in PbS, the inferred single band effective mass is therefore $m^*_b \approx 0.19$ whereas it is expected to be closer to 0.15 at 800 K^21^. This discrepancy is also confirmed if the figure of merit is plotted as a function of the charge carriers density (not shown here) with the aforementioned set of parameters B and $m^*_d$. Whereas the agreement with the experimental data is actually very good above the maximum of ZT, the expected variation overestimates the figure of merit below namely for low charge carriers density. This result might suggest that this regime is additionally influenced by the valence bands contribution which implies bipolar effects and then reduces the thermoelectric efficiency. Therefore, the extension of the presently reported formalism to the case of multiple bands including bipolar effects should fix this issue by allowing to account for the observed figure of merit in such materials. Another example is discussed in the Supplementary information.

Furthermore, the expression of $N_c(T,\gamma =1/2)$ shows due to the considered free electrons density of states an effective mass dependence as $m^{3/2}$. A low band effective mass $m^*_b$ is therefore expected to decrease the charge carriers density and shifts the ZT maximum towards lower n values without altering its amplitude as illustrated in Fig. 3(a) for a constant B. Nevertheless, the material quality factor is also expected to depend on the effective mass according the electrical conductivity parameter $\sigma _{E_0}= q^2 \frac{\tau _0 v_0^2 g_0}{E_{\tau }^\theta E_{v}^ \nu E_{g}^\gamma } (k_B T)^s$, with characteristic energies $E_{\tau }, E_{v}$ and $E_g$ and the constants $\tau _0, v_0$ and $g_0$ as previously introduced^18^. If one considers the $\hbox {s}=1$ case with $\gamma =1/2$, $\theta =-1/2$ and $\nu =1$, one recovers the previously mentioned dependence of the density of states with $g_0/ E_{g}^{1/2} \propto (m^*_b) ^{3/2}$ while due to the acoustic phonon scattering $\tau _0/ E_{\tau }^{-1/2} \propto (m^*_b)^{-3/2}$. Thus, the remaining dependence originates from the velocity term $v_0^2/E_{v} \propto m_I^{-1}$, with $m_I$ which is usually referred as an inertial effective mass and defined by Goldsmid^9^ as $m_I=3 (1/m_1+1/m_2+1/m_3)^{-1}$ where $m_i$ are the principal values of the effective mass when the equal energy surfaces are ellipsoids. In such a case, the band effective mass is therefore $m^*_b = (m_1 m_2 m_3)^{1/3}$. For an isotropic system with $m^*_b = m_I = m^*$, it follows that $\sigma _{E_0} \propto 1/m^*$ and then that $B \propto 1/m^*$.

It results in this case that a low effective mass is indeed beneficial to the figure of merit as illustrated in Fig. 3(b) since the decrease of $m^*$ not only shifts the ZT maximum down to lower charge carriers density but also increases the value of the material quality factor B and then ZT.

### Transport coefficients in the intermediate regime

On the other hand, the reformulation of ZT in Eq. (9) can provide new insight from an analytical point of view if one considers the limit of zero chemical potential or the equivalent limit of infinite temperature. By using the property of the Polylogarithm $Li_s(-1)=- \eta _s$, with the Dirichlet $\eta$ function $\eta _s= \sum _{k=1}^\infty \frac{(-1)^{k-1}}{k^{s}}$, the exact expressions of the transport coefficients can be given whatever s for $\tilde{\mu } = 0$.

11 $$\begin{aligned} \frac{\sigma }{\sigma _{E_0}}_{\tilde{\mu } = 0}= &\; {} \Gamma (s+1) \eta _s \;\;\;\;\; \;\;\;\;\; \alpha _{\tilde{\mu } = 0} = \frac{k_B}{q} (s+1)\frac{\eta _{s+1}}{\eta _s} \;\;\;\;\; \;\;\;\;\; \nonumber \\ L_{\tilde{\mu } = 0}= &\; {} \left( \frac{k_B}{q} \right) ^2 \frac{(s+1)}{\eta _s} \left[ (s+2) \eta _{s+2} - (s+1) \frac{\eta ^2_{s+1}}{\eta _s} \right] \end{aligned}$$

These relations demonstrate that the asymptotic behavior of the transport coefficients is fully governed by the energy dependence in the transport integrals, namely by the exponent s which originates from the energy dependence of the relaxation time, the quasiparticles velocity and the density of states. As a consequence, it results that the high temperatures values of the electrical conductivity, the thermopower and the Lorentz number can provide an interesting way to determine experimentally the exponent s, and then to characterize the microscopic energy dependence in the transport integrals. According to Eq. (9), the figure of merit can also be simply written in this limit as a function of B, s and the Dirichlet $\eta$ function.

12 $$\begin{aligned} ZT_{\tilde{\mu } = 0} = \left[ \left( \frac{s+2}{s+1} \right) \frac{\eta _s \eta _{s+2}}{ \eta _{s+1}^2} + \frac{\eta _s}{ (s+1) B \Gamma _{s+2} \eta _{s+1}^2} -1 \right] ^{-1} \end{aligned}$$

**Figure 4:**
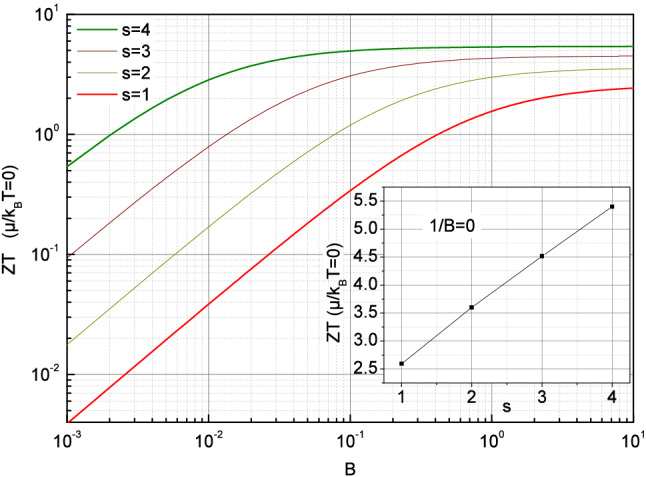
Variation of the figure of merit with $\tilde{\mu } = 0$ as a function of the material quality factor *B* for $\hbox {s}=1$, 2, 3 and 4. The inset shows the figure of merit for $\tilde{\mu } = 0$ as a function of s when $1/B=0$ namely without the lattice contribution to the thermal conductivity.

This relation leads therefore to the variations of the figure of merit shown in Fig. 4 as a function of the material quality factor for the exponents s ranging from 1 up to 4.

In connection with Fig. 1, the variations displayed in Fig. 4 highlight the large values of figure of merit which can be reached in this regime as well as the influence of the exponent s on the material quality factor required to exceed $ZT=1$. It also illustrates that the asymptotic behavior shown in the inset of Fig. 4 without the lattice contribution to the thermal conductivity appears much more easily achieved with high exponents s, namely for lower material quality factor. While the limit $\tilde{\mu } = 0$ may appear rather theoretical, the relation 10 allows to precise the corresponding charge carriers density $n_{\tilde{\mu } = 0} = \eta _{\gamma +1} N_c(T, \gamma )$ which yields nearly to 2 $10^{25}\,\hbox {m}^{-3}$ for $\gamma =1/2$ at 300 K without effective mass renormalization as shown in Fig. 2(a).

Even if large figure of merit can be reached in this regime it does not correspond to its maximum value which departs slightly from $\tilde{\mu }=0$ depending on s and B as shown in Fig. 1. It is therefore interesting to better characterize the regime around this limit where basically $| \tilde{\mu } | <1$, and which appears intermediate between the strongly degenerate regime and the completely non-degenerate one. Whereas approximations already exist for the two latter regimes, there are none for this intermediate one. One strategy consists thus to expand the Fermi integral in power series as a function of $\tilde{\mu }$ around $\tilde{\mu }=0$. In order to perform this, the definition of the Polylogarithm function may be reformulated by expanding its argument as below.

$$\begin{aligned} Li_{s+1}(- e^{ \tilde{\mu }} )= &\; {} \sum _{k=1}^\infty \frac{(-1)^{k}}{k^{s+1}} e^{k \tilde{\mu } } = - \sum _{k=1}^\infty \frac{(-1)^{k-1}}{k^{s+1}} \sum _{m=0}^\infty \frac{(k \tilde{\mu })^m }{m !} = -\sum _{m=0}^\infty \sum _{k=1}^\infty \frac{(-1)^{k-1}}{k^{s+1-m}} \frac{\tilde{\mu }^m }{m !} \\= &\; {} - \sum _{m=0}^\infty \eta _{s+1-m} \frac{\tilde{\mu }^m }{m !} \end{aligned}$$

The Dirichlet $\eta$ function has here been recognized with $\eta _{s+1-m} = \sum _{k=1}^\infty \frac{(-1)^{k-1}}{k^{s+1-m}}$. Some values of the Dirichlet $\eta$ function which cover the range of interest if the exponent s involved in the Fermi integrals varies from 1 up to 4 are provided in the Supplementary information.

Thus, it follows that the Fermi integrals can be expanded in power series as a function of $\tilde{\mu }$ such as:

13 $$\begin{aligned} F_s( \tilde{\mu } ) = \Gamma (s+1)\sum _{m=0}^\infty \eta _{s+1-m} \frac{\tilde{\mu }^m }{m !} \approx \Gamma (s+1) \left( \eta _{s+1} +\eta _{s}\tilde{\mu } +\eta _{s-1} \frac{\tilde{\mu }^2 }{2} \right) \end{aligned}$$

Note that one recovers in Eq. (13) the property of the Polylogarithm $Li_s(-1)=- \eta _s$ as previously used to investigate the limit $\tilde{\mu }=0$. The use of the expansion 13 allows now to deduce the expressions of the electrical conductivity and the thermopower in the intermediate regime according their definition up to second order.

14 $$\begin{aligned} \frac{\sigma }{\sigma _{E_0}}\approx  \Gamma (s+1) \left( \eta _s +\eta _{s-1}\tilde{\mu } +\eta _{s-2} \frac{\tilde{\mu }^2 }{2} \right) \end{aligned}$$

15 $$\begin{aligned} \alpha\approx &  \alpha _0 \left( 1 +\tilde{\mu } \left[ \frac{s}{(s+1)}\frac{\eta _{s}}{\eta _{s+1}}-\frac{\eta _{s-1}}{\eta _{s}} \right] -\frac{\tilde{\mu }^2 }{2} \left[ \frac{\eta _{s-1}}{\eta _{s+1}} + \frac{\eta _{s-2}}{\eta _{s}} -2 \frac{\eta ^2_{s-1}}{\eta ^2_{s}} \right] \right) \nonumber \\&\quad with\quad \alpha _0 = \frac{k_B}{q} (s+1)\frac{\eta _{s+1}}{\eta _s} \end{aligned}$$

**Figure 5:**
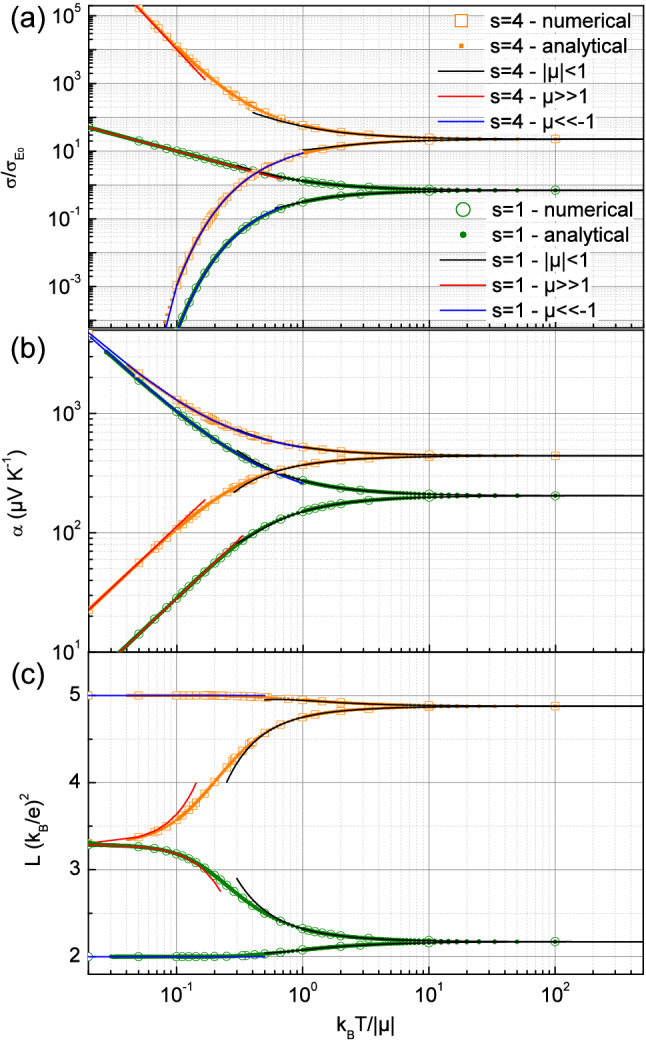
(**a**) Normalized electrical conductivity $\sigma /\sigma _{E_0}$, (**b**) absolute value of the thermopower $\alpha$ and (**c**) Lorentz number in unit of $(k_B/e)^2$ as a function of the reduced temperature $k_B T/|\mu |$ for $\hbox {s}=1$ and 4. Here the three kinds of expansion corresponding to the regimes $|\tilde{\mu }|<1$, $\tilde{\mu }<<-1$ and $\tilde{\mu }>>1$ are compared to numerical and analytical results.

These relations can be compared in Fig. 5(a) and (b) with the electrical conductivity and the thermopower as deduced from numerical computation of the required Fermi integrals or from direct analytical calculations with Eqs. (5) and (6) for $\hbox {s}=4$ and 1. It turns out that the agreement is quite satisfactory especially when $\tilde{\mu }$ is small, or in the limit of high temperatures, but also up to nearly $|\tilde{\mu }| < 2$ due to the second order corrections.

On the other hand, the Lorentz number can also be expanded with Eq. (13) as $L \approx \left( \frac{k_B}{q} \right) ^2 \left( l_0 + l_1 \tilde{\mu } + l_2 \tilde{\mu }^2 \right)$ by introducing the dimensionless coefficients $l_i$ with $L_{\tilde{\mu } = 0}= (k_B/q)^2 l_0$, the full expressions of $l_1$ and $l_2$ being given in the Supplementary information. The Lorentz number can thus be compared in Fig. 5(c) with numerical results for $s=1$ and 4 when plotted as a function of the reduced temperature. As previously suggested, Fig. 5(a), (b) and (c) clearly illustrate that the asymptotic behavior of the electrical conductivity, the thermopower and the Lorentz number here reached when $k_B T > 10\,\mu$ are entirely governed by the exponent s. In particular, the three asymptotic regimes of the Lorentz number are clearly displayed in Fig. 5(c) with the value $l_0$ given by Eq. (11) when $k_B T > 10\, \mu$ (intermediate regime), the recovered Wiedmann–Franz law with the limit $\pi ^2 /3$ reached whatever s when $k_B T<< \mu /10$ (highly degenerate regime), and the value $(s+1)$ according Eq. (8) when $k_B T<< -\mu /10$ (strongly non-degenerate regime). It appears also that the intermediate regime power expansion can help in determining experimentally the exponent s in a lower and then more accessible temperature range such as $k_B T > \mu / 2$.

Furthermore, Eq. (12) provides also the zero order term $z_0$ of the power expansion of the figure of merit which can be written up to second order as below.

16 $$\begin{aligned} ZT_{| \tilde{\mu } | <1} \approx z_0 + z_1 \tilde{\mu } + z_2 \tilde{\mu }^2 \end{aligned}$$

$z_0$ is defined according to Eq. (12) and the other coefficients $z_1$ and $z_2$ can be expressed as a function of $z_0$ as shown in the Supplementary information. According to Eq. (16), it results that the optimal figure of merit is reached when $\tilde{\mu }_{ZT_{max}}=-z_1/(2z_2)$ which leads to the maximum $ZT_{max}=z_0-z_1^2/(4z_2)$. By using the same procedure, the charge carriers density can finally be expanded up to second order and the chemical potential can be related to n as it follows.

17 $$\begin{aligned} n \approx n_{\tilde{\mu } = 0} \left( 1 +\frac{\eta _{\gamma }}{\eta _{\gamma +1}} \tilde{\mu } +\frac{\eta _{\gamma -1}}{\eta _{\gamma +1}} \frac{\tilde{\mu }^2 }{2} \right) \qquad \qquad with \qquad \qquad n_{\tilde{\mu } = 0} = \eta _{\gamma +1} N_c(T, \gamma ) \end{aligned}$$

The latter expansion allows then to infer the charge carriers density for $\tilde{\mu }_{ZT_{max}}$ by fully characterizing the maximum of the figure of merit in this regime.

So, whereas the thermoelectric transport coefficients are usually defined as a function of Fermi integrals, it is shown here that their explicit calculation leads to reformulate these coefficients by introducing Polylogarithm functions. This provides in particular a new definition of the figure of merit which covers all the regimes of interest including the intermediate one. While this formalism is found to reproduce quite well the figure of merit measured experimentally in the PbS compound by simply adjusting the material quality factor and the effective mass, it should allow a straightforward extension to more complex materials such as other lead chalcogenides which require to take into account multiple bands without implying numerical calculations. In addition, this formulation of the Fermi integrals provides a good starting point in order to perform a power expansion which leads to a new approximation specifically relevant for the intermediate regime connecting thus the highly degenerate to the strongly non-degenerate ones. It turns out that the transport coefficients can then be expanded in the frame of this approximation which reveals their asymptotic behaviors in the limit of high temperatures. These results shed new light on the thermoelectric transport properties of the materials and point out that the analysis of their high temperatures behaviors can allow to characterize experimentally the microscopic energy dependence in the transport integrals.

## Supplementary Information

Supplementary Information.
