# Supplementary material for: Thermopower, figure of merit and Fermi integrals
Source: Sci Rep. 2021 Dec 21;11:24323. doi: 10.1038/s41598-021-03760-4 (PMC8692468; doi:10.1038/s41598-021-03760-4)
Supplement: Supplementary file 1 — Supplementary Information. [file 41598_2021_3760_MOESM1_ESM.pdf]

Supplementary information

Thermopower, figure of merit and Fermi integrals

Patrice Limelette[1,*]

[1]GREMAN, UMR 7347 CNRS-INSA-Université de Tours, Parc de Grandmont, 37200 TOURS, FRANCE
[*]patrice.limelette@univ-tours.fr

ABSTRACT

As explained in the main text the transport coefficients characterizing the thermoelectric figure of merit can be written as a function of Fermi integrals which can be calculated as a function of Polylogarithm. Below, an alternative demonstration is provided without expanding the Fermi factor but instead by using the recurrence formula which relates one Fermi integral to another. On the other hand, the expansions coefficients of the electrical conductivity, the thermopower, the Lorentz number and the figure of merit are here explicitly detailled up to second order.

Fermi integrals from the recurrence formula

The electrical conductivity, the thermopower, the Lorentz number and the figure of merit can be defined at a semi-classical level as a function of Fermi integrals.[1–3] The calculation of these Fermi integrals $F_s(\tilde{\mu})$ can be carried out by expanding the Fermi factor as demonstrated in the main text but also with the use of the so-called recurrence formula which relates $F_{s+1}(\tilde{\mu})$ to $F_s(\tilde{\mu})$. As already reported by Fistul,[4] the latter formula can be demonstrated by considering the two following relations with $A = e^{\tilde{\mu}}$.

$$\partial_A F_{s+1}(\tilde{\mu}) = \int_0^\infty \frac{x^{s+1}A^{-2}e^x}{(A^{-1}e^x+1)^2}dx \qquad and \qquad \partial_x \left(A^{-1}e^x+1\right)^{-1} = \frac{-A^{-1}e^x}{(A^{-1}e^x+1)^2}$$

The combination of these two relations allows then to relate $F_{s+1}(\tilde{\mu})$ to $F_s(\tilde{\mu})$ through a straightforward integration by parts such as:

$$\begin{aligned}\partial_A F_{s+1}(\tilde{\mu}) &= \int_0^\infty x^{s+1}A^{-1}\partial_x\left(A^{-1}e^x+1\right)^{-1}dx = -[x^{s+1}A^{-1}\left(A^{-1}e^x+1\right)^{-1}]_0^\infty + (s+1)\int_0^\infty x^sA^{-1}\left(A^{-1}e^x+1\right)^{-1}dx \\ &= (s+1)\int_0^\infty x^sA^{-1}\left(A^{-1}e^x+1\right)^{-1}dx = (s+1)A^{-1}F_s(\tilde{\mu})\end{aligned}$$

Therefore, the following integral recurrence formula can be inferred.

$$F_{s+1}(\tilde{\mu}) = \int_0^A (s+1)A^{-1}F_s(\tilde{\mu})dA$$

On the other hand, the Fermi integral $F_0(\tilde{\mu})$ can be exactly calculated by involving a logarithmic form as well known.

$$F_0(\tilde{\mu}) = \int_0^\infty \frac{1}{e^{x-\tilde{\mu}}+1}dx = \int_0^\infty \frac{e^{-(x-\tilde{\mu})}}{1+e^{-(x-\tilde{\mu})}}dx = ln\left(1+e^{\tilde{\mu}}\right) = ln\left(1+A\right)$$

Now, the previous recurrence formula can be used to calculate $F_1(\tilde{\mu})$ by expanding $F_0(\tilde{\mu})$ in power series.

$$F_1(\tilde{\mu}) = \int_0^A A^{-1}F_0(\tilde{\mu})dA = \int_0^A A^{-1}ln\left(1+A\right)dA = \int_0^A A^{-1}\sum_{k=1}^\infty \frac{(-1)^{k-1}}{k}A^k dA = \sum_{k=1}^\infty \frac{(-1)^{k-1}}{k}\int_0^A A^{k-1}dA = \sum_{k=1}^\infty \frac{(-1)^{k-1}}{k^2}A^k$$

As previously introduced, one recovers here the power expansion of a dilogarithm $Li_2(-A)$. By using the same procedure, the Fermi integral $F_2(\tilde{\mu})$ can be determined from $F_1(\tilde{\mu})$, $F_3(\tilde{\mu})$ from $F_2(\tilde{\mu})$ and so on.

$$F_2(\tilde{\mu}) = 2\int_0^A A^{-1}F_1(\tilde{\mu})dA = 2\sum_{k=1}^\infty \frac{(-1)^{k-1}}{k^2}\int_0^A A^{k-1}dA = 2\sum_{k=1}^\infty \frac{(-1)^{k-1}}{k^3}A^k \qquad \Rightarrow \qquad F_3(\tilde{\mu}) = 3!\sum_{k=1}^\infty \frac{(-1)^{k-1}}{k^4}A^k$$

Thus, the Fermi integral $F_2(\tilde{\mu})$ implies a trilogarithm function and the expression of $F_3(\tilde{\mu})$ allows to generalize the result for an arbitrary Fermi integral such as $F_s(\tilde{\mu}) = -\Gamma(s+1)Li_{s+1}(-e^{\tilde{\mu}})$ in agreement with the relation previously demonstrated in the main text.

Transport coefficients in the intermediate regime

The use of the Fermi integral expansion $F_s(\tilde{\mu}) = \Gamma(s+1)\sum_{m=0}^{\infty}\eta_{s+1-m}\frac{\tilde{\mu}^m}{m!}$ allows to deduce the expressions of the electrical conductivity and the thermopower in the intermediate regime according their definition up to second order as raised in the main text.

$$\frac{\sigma}{\sigma_{E_0}} \approx \Gamma(s+1)\left(\eta_s + \eta_{s-1}\tilde{\mu} + \eta_{s-2}\frac{\tilde{\mu}^2}{2}\right) \tag{1}$$

$$\alpha \approx \alpha_0\left(1+\tilde{\mu}\left[\frac{s}{(s+1)}\frac{\eta_s}{\eta_{s+1}} - \frac{\eta_{s-1}}{\eta_s}\right] - \frac{\tilde{\mu}^2}{2}\left[\frac{\eta_{s-1}}{\eta_{s+1}} + \frac{\eta_{s-2}}{\eta_s} - 2\frac{\eta_{s-1}^2}{\eta_s^2}\right]\right) \quad with \quad \alpha_0 = \frac{k_B}{q}(s+1)\frac{\eta_{s+1}}{\eta_s} \tag{2}$$

In addition, the Lorentz number can also be expanded up to second order as it follows by introducing the dimensionless coefficients l_i with $L_{\tilde{\mu}=0} = (k_B/q)^2 l_0$.

$$L \approx \left(\frac{k_B}{q}\right)^2 \left(l_0 + l_1\tilde{\mu} + l_2\tilde{\mu}^2\right) \tag{3}$$

$$l_0 = \frac{(s+1)}{\eta_s}\left[(s+2)\eta_{s+2} - (s+1)\frac{\eta_{s+1}^2}{\eta_s}\right]$$

$$l_1 = \frac{(s+1)}{\eta_s}\left[2(s+1)\eta_{s-1}\frac{\eta_{s+1}^2}{\eta_s^2} - s\eta_{s+1} - (s+2)\frac{\eta_{s-1}\eta_{s+2}}{\eta_s}\right]$$

$$l_2 = \frac{(s+1)}{\eta_s}\left[-\frac{s}{2}\eta_s + (2s+1)\frac{\eta_{s-1}\eta_{s+1}}{\eta_s} + (s+2)\frac{\eta_{s+2}}{\eta_s}\left(\frac{\eta_{s-1}^2}{\eta_s} - \frac{\eta_{s-2}}{2}\right) + (s+1)\frac{\eta_{s+1}^2}{\eta_s^2}\left(\eta_{s-2} - 3\frac{\eta_{s-1}^2}{\eta_s}\right)\right]$$

As emphasized in the main text, the expansions 1, 2 and 3 reproduce quite well the expected variations of the electrical conductivity, the thermopower and the Lorentz number in a high temperatures regime such as $k_BT > \mu/2$ due to the second order correction.

It is worth mentioning that even if the latter expressions could appear rather complicated, they are easily simplified for a given s by using the corresponding values of the Dirichlet η function. For this purpose, some relevant values are indicated in the Table 1 which cover the range of interest if the exponent s involved in the Fermi integrals varies from 1 up to 4.

s	-1	0	1	2	3	4	5	6
η_s	1/4	1/2	$ln(2)$	$\pi^2/12$	$3/4\,\zeta(3)$	$7\pi^4/720$	$15/16\,\zeta(5)$	$31\pi^6/30240$
ζ_s	$-1/12$	$-1/2$	∞	$\pi^2/6$	1.20206	$\pi^4/90$	1.03693	$\pi^6/945$

Table 1. Selected values of the Dirichlet η and Riemann ζ functions. Note that both functions are related as $\eta_s = (1-2^{1-s})\zeta_s$ when ζ is well defined.

Furthermore, the figure of merit can finally be expanded such as $ZT_{|\tilde{\mu}|<1} = z_0 + z_1\tilde{\mu} + z_2\tilde{\mu}^2$, with the zero order term $z_0 = ZT_{\tilde{\mu}=0}$ introduced in the main text and the other coefficients z_1 and z_2 defined below.

$$z_1 = \left(\frac{2s\eta_s}{(1+s)\eta_{s+1}} - \frac{\eta_{s-1}}{\eta_s}\right)z_0 + \left(\frac{s\eta_s}{(s+1)\eta_{s+1}} - \frac{\eta_{s-1}}{\eta_s}\right)z_0^2$$

$$z_2 = \left(\frac{\eta_{s-1}^2}{\eta_s^2} - \frac{\eta_{s-1}}{\eta_{s+1}} - \frac{\eta_{s-2}}{2\eta_s} + \frac{s^2\eta_s^2}{(s+1)^2\eta_{s+1}^2}\right)z_0 - \left(\frac{\eta_{s-2}}{2\eta_s} - \frac{2\eta_{s-1}^2}{\eta_s^2} - \frac{s\eta_s^2}{2(s+1)\eta_{s+1}^2} + \frac{(4s+1)\eta_{s-1}}{(s+1)\eta_{s+1}} - \frac{2s^2\eta_s^2}{(s+1)^2\eta_{s+1}^2}\right)z_0^2 + \left(\frac{\eta_{s-1}}{\eta_s} - \frac{s\eta_s}{(s+1)\eta_{s+1}}\right)^2 z_0^3$$

These coefficients allow to characterize the maximum of the figure of merit in the intermediate regime, the related chemical potential and then the charge carriers density.

Skutterudites example

In order to illustrate the applicability of the reported formalism to thermoelectric materials other than chalcogenides, the case of the skutterudites $CoSb_3$ can be considered for instance. The n-type filled skutterudites are actually among the best thermoelectric materials. The addition of filler atoms such as Yb into a void site can lead to high ZT by reducing the thermal conductivity while simultaneously doping the material by adding electrons as charge carriers. Besides, the skutterudites are known to have a favorable electronic band structure ascribed to threefold degeneracy at the conduction band minimum, and it has been shown quite recently that a secondary conduction band with 12 carrier pockets converges with the primary band at high temperatures by explaining their extraordinary thermoelectric performance.[5] These characteristics imply then that a rigorous treatment of the transport properties requires to take into account these multiple bands. Therefore, a numerical three bands description has been proposed,[5] by involving two conduction and one valence bands to account for the highest thermoelectric performance measured at 800 K. As a consequence, an extension of the analytical formalism reported here is required in order to be applied to these materials in such a condition. Nevertheless, if one considers the thermoelectric performance measured at 300 K in the skutterudites $Yb_xCo_4Sb_{12}$ when the second conduction band has not yet converged, an effective one-band description can be attempted.

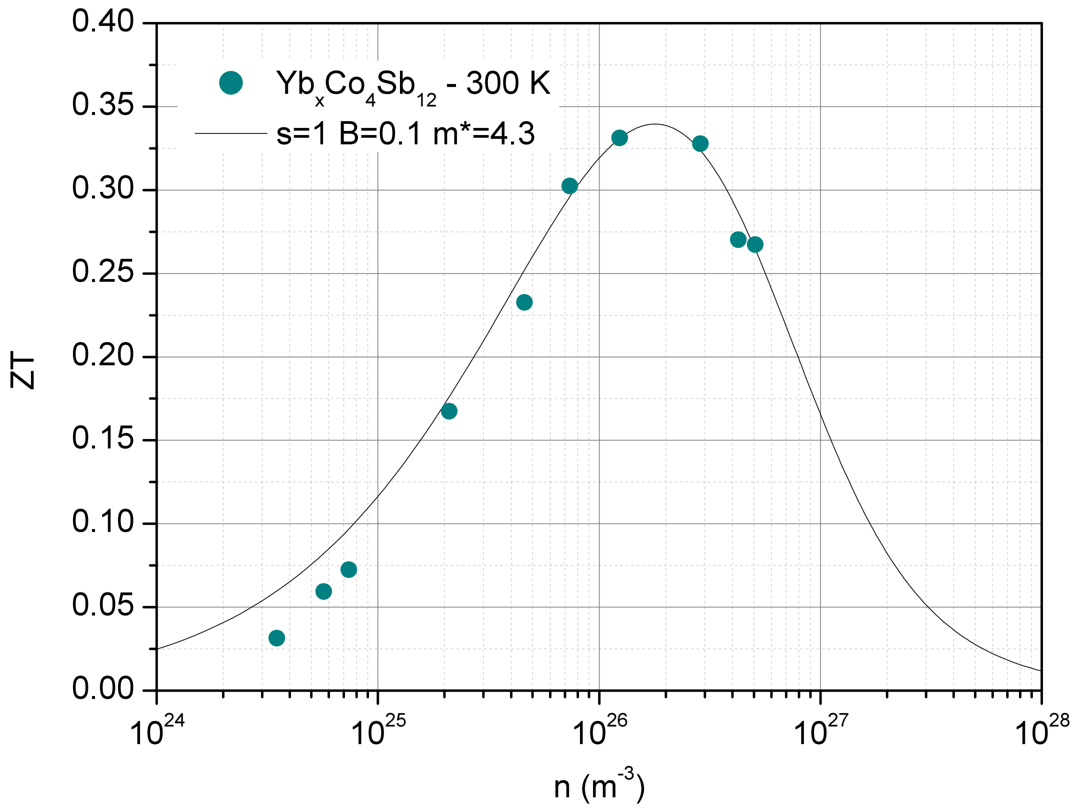

Figure 1. Comparison between the figure of merit determined experimentally in the n-type Skutterudite at T=300 K with the one calculated with s=1 and $\gamma = 1/2$. Note that the experimental data have been taken from Fig. S4 of the related reference.[5]

As shown in Fig. 1, the variation of the figure of merit determined experimentally in these Skutterudites as a function of the charge carriers density is rather well reproduced at 300 K even if one must keep in mind that a rigorous treatment would necessitate to take into account the multiple bands structure. While this prevents from interpreting quantitatively the found density of states effective mass $m^* \approx 4.3$, this high value points out a large valley degeneracy in a qualitative agreement with their electronic structure.

By demonstrating that the reported formalism is not restricted to chalcogenides but could be in principle applied to a wide variety of thermoelectric materials, this comparison emphasizes the interest to extend the reported formalism to the case of multiple bands materials.

References

1. Chasmar, R. P. & Stratton, R. The Thermoelectric Figure of Merit and its Relation to Thermoelectric Generators. *J. Electron. Control.* **7**, 52-72, DOI: https://doi.org/10.1080/00207215908937186 (1959).

2. Testardi, L. R. Calculations of the Thermoelectric Parameters and the Maximum Figure of Merit for Acoustical Scattering. *J. Appl. Phys.* **32**, 1978, DOI: https://doi.org/10.1063/1.1728275 (1961).

3. Goldsmid, R. P., *Thermoelectric Refrigeration* (Plenum Press), DOI: https://doi.org/10.1007/978-1-4899-5723-8 (1964).

4. Fistul, V. I. *Heavily doped semiconductors* (Plenum Press), DOI: https://doi.org/10.1007/978-1-4684-8821-0 (1969).

5. Tang, Y., Gibbs, Z. M., Agapito, L. A., Li, G., Kim, H-S., Nardelli, M. B., Curtarolo, S. & Snyder, G. J. Convergence of multi-valley bands as the electronic origin of high thermoelectric performance in CoSb3 skutterudites. *Nat. Mater.* **14**, 1223–1228, DOI: https://doi.org/10.1038/nmat4430 (2015).
